# Rethinking trazodone for insomnia in alcohol use disorder

**DOI:** 10.1186/s13722-025-00552-3

**Published:** 2025-03-25

**Authors:** Jeffrey Pan, Jürgen Rehm, Evan Wood

**Affiliations:** 1 British Columbia Centre on Substance Use, Vancouver, Canada; 2 Faculty of Medicine, University of British Columbia, Vancouver, Canada; 3 Institute for Mental Health Policy Research & Campbell Family Mental Health Research Institute, Centre for Addiction and Mental Health (CAMH), Toronto, Canada; 4 Dalla Lana School of Public Health, Institute of Health Policy, Management and Evaluation & Department of Psychiatry, University of Toronto (UofT), Toronto, Canada; 5 Zentrum für Interdisziplinäre Suchtforschung, Universität Hamburg (ZIS), Universitätsklinikum Hamburg-Eppendorf, Hamburg, Germany; 6 Canada Research Chair in Addiction Medicine, University of British Columbia, 4th Floor, 1045 Howe Street, V6Z 2A9 Vancouver, BC Canada

**Keywords:** Alcohol use disorder, Trazodone, Alcohol craving, Meta-Chlorophenylpiperazine, Polypharmacy

## Abstract

**Background:**

Insomnia is a common condition experienced by many individuals with excessive alcohol use and alcohol use disorder, and the serotonin antagonist and reuptake inhibitor trazodone has emerged as a mainstay of treatment for insomnia in this population.

**Main body:**

However, an underappreciated literature has demonstrated potential for an increase in alcohol use while persons with alcohol use disorder are taking trazodone for sleep challenges. Additionally, multiple trials have identified trazodone’s metabolite meta-Chlorophenylpiperazine as a pharmaceutical inducer of increased alcohol craving and use.

**Conclusion:**

Increased awareness in the potential of worsening drinking behaviour with trazodone accompanied by the preferential use of safer alternative treatment strategies can likely improve outcomes for patients with heavy drinking and alcohol use disorder.

## Background

Insomnia and complaints of poor sleep are a common presentation among individuals with excessive alcohol use and alcohol use disorder, which can significantly hinder the effectiveness of treatment efforts. For instance, among individuals with alcohol use disorder, studies have suggested that greater than 50% of inpatients being treated for alcohol use disorder reported insomnia in early abstinence and a majority reporting insomnia for more than a year prior [1]. Alcohol use disorder involves numerous factors that contribute to insomnia, including withdrawal symptoms, disruption of sleep architecture, and co-occurring mental health challenges such as depression [1], Certain medications used for the treatment of alcohol use disorder may also contribute to sleep challenges [2]. Outside of alcohol use disorder, studies have suggested that as many as 75% of individuals who use alcohol excessively may complain of poor sleep [3, 4].

## The evidence

Multiple psychological treatment and pharmacologic approaches have emerged for the treatment of insomnia including cognitive behavioral therapy, the use of sedative-hypnotic medications as well as various off-label medications [5]. Due to potential for tolerance and dependence, sedative-hypnotics are generally used with caution in the general population, and often avoided in individuals with alcohol use disorder due to the increased risk of abuse and other harms [6].

Among the off-label medications used for insomnia, the serotonin antagonist and reuptake inhibitor trazodone has sedating qualities as part of its side-effect profile and has been one of the most used prescription medications for insomnia in the general population and among individuals with alcohol use disorder [7, 8]. Among patients with alcohol use disorder, a U.S. random systematic sample of addiction medicine physicians’ medical management of sleep disturbance among patients in early recovery from alcoholism found that trazodone was the preferred medication of choice “despite limited evidence for or against this indication” [7]. Outside of patients with alcohol use disorder, in a clinical suitability appraisal study focusing on the question of “should trazodone be first-line therapy for insomnia,” it was found that a panel of key opinion leaders supported trazodone as the first-line agent [8]. Similarly, a meta-analysis of randomized trials published in 2022 has provided cautious support for trazodone in insomnia disorders concluding that “trazodone could improve sleep by changing the sleep architecture in insomnia disorder, but it should be used with caution due to the adverse events that may occur” [9].

A systematic review of pharmacological agents that could treat sleep problems for individuals in alcohol recovery found that trazodone has the most data suggesting possible efficacy, but the finding is tempered by a study suggesting its association with heavier alcohol use [10]. In this context, polypharmacy is a common occurrence in individuals with alcohol use disorder leading to the common use of medications that have had benefits demonstrated in trials that exclude persons with alcohol use disorder. For instance, a review of 170 antidepressant efficacy trials demonstrated routine exclusion of patients with substance use disorder and raised “the question of whether the routine exclusion of patients with a substance use disorder should be reflected in a product’s label” [11].

In this context, few studies have examined the effects of trazodone among persons with alcohol use disorder. One prospective trial by Friedmann et al. [12] randomized 173 individuals during alcohol detoxification to oral trazodone (50-150 mg) versus placebo and reported that the trazodone group experienced less improvement in days abstinent during the three month trial [7]. Interestingly, the effect of increased drinking persisted for three months after the medication was stopped in comparison to the placebo group, an apparent drug effect also seen in a trial of the SSRI sertraline [13]. Although patients randomized to trazodone were more likely to experience improvement in their sleep quality, these investigators concluded that the use of this medication may lead to an increase in alcohol consumption in the post detoxification period [7]. In another randomized placebo-controlled double-blind study, Wetzel et al. [14] examined at the effects of nefazodone, an analogue of trazodone, on relapse prevention in alcohol-dependent men at three German university centres [14]. Similar to the trial by Friedmann and colleagues, these investigators concluded that nefazodone may increase the amount of alcohol consumed per relapse in comparison to placebo [14].

Findings from observational studies of trazodone in persons with alcohol use have been mixed. Monnelly et al. [15] used a national administrative data from the Department of Veterans Affairs, to identify 14,443 hospitalizations and compare the differences in time to rehospitalization after discharge from an index hospitalization for alcohol dependence and found an increased risk of rehospitalization among those prescribed trazodone in isolation, a finding most evident in those with 2 or more previously discharges [15]. A single site retrospective study (*n* = 283) examined self-reported relapse via responses to a mail in survey (supplemented by telephone contact where possible) following admission to a residential treatment center, and reported no association between trazodone use and relapse rates at 6 months though only 37 relapses were disclosed among those providing self-reported data [16].

The use of trazodone in an effort to improve sleep paradoxically increasing increase alcohol use may be a source of confusion to some prescribers but may have a clear biological basis. For instance, studies have implicated the serotonergic system in alcohol use disorder and, where trazodone’s metabolite meta-Chlorophenylpiperazine (m-CPP) has been studied, it has demonstrated potential for increased alcohol craving and alcohol use despite small sample size trials at high risk of Type 2 error. For instance, Benkelfat et al. [17] administered saline followed by 0.8 mg/kg m-CPP to 21 patients with alcohol use disorder after three weeks of abstinence and found that eleven patients reported a “high” feeling comparable to the effect of alcohol after m-CPP administration and that seven patients reported “an urge (craving) to use alcohol while the placebo did not” [17]. Similarly, Krystal et al. [18] randomized 22 male individuals to 0.1 mg/kg m-CPP infusion, 0.4 mg/g yohimbine infusion or saline infusion and reported that “m-CPP but not saline significantly increased craving for alcohol” [18]. While the findings of a randomized trial of 16 individuals to oral 0.5 mg/kg m-CPP vs. placebo by Buydens-Branchey et al. [19] were inconsistent with these initial reports, the investigators reported a significantly more intense “high” feeling after m-CPP when alcohol was used in comparison to placebo [19]. In the most recent double-blind, placebo-control trial, Umhau et al. [20] randomized 35 treatment seeking alcohol dependent inpatients to saline, or “pharmacologically induced alcohol craving” with m-CPP or yohimbine infusion following 2 weeks of placebo or acamprosate use for the treatment of alcohol dependency. Here, the investigators reported that “cravings were modestly, but significantly higher following m-CPP challenge compared with saline infusion” and that “alcohol cravings induced by these two stimuli are not sensitive to acamprosate at clinically used doses” [20]. Consistent with the above trial of the trazodone analogue nefazodone, we note that m-CPP is also a psychoactive metabolite of this medication potentially explaining heavier drinking in comparison to placebo in this study [14].

Importantly, these findings are not unique to serotonergic system stimulation with trazodone or m-CPP. For instance, as summarized elsewhere [21], a number of double-blind placebo-controlled trials have demonstrated increased drinking among some patients with alcohol use disorder prescribed selective serotonin re-uptake inhibitors. While some individual trials have suggested certain benefits of serotonergic antidepressants in substance use disorders, similar results have been reported for increased tobacco [22, 23], cocaine [24, 25], methamphetamine [26, 27] and cannabis use [28] in trials of certain serotonergic antidepressants. In this context, it is noteworthy that for on-label indications (e.g. anxiety, depression), the evidence for serotonergic antidepressants in alcohol use disorder is poor [29, 30].

The relationship between alcohol use and insomnia, as well as the improvements in insomnia symptoms with a reduction in alcohol use or abstinence, are well described [31, 32]. Here, investigators have concluded that preventing relapse to heavy drinking provides good first line therapy for insomnia in AUD patients [32]. In this context, a systematic review conducted by Panin & Peana [2] found that disulfiram and naltrexone may have a negative effect on sleep, whereas acamprosate may provide benefits to sleep architecture [2]. Additionally, while overall less researched than other AUD pharmacotherapies [2], gabapentin may have benefits for both alcohol use disorder and insomnia in this population [33]. The above findings speak to the potential of a goal directed pharmacotherapy approach based on patient goals and relevant comorbidities [34]. While more fulsome reviews of additional pharmacotherapies (e.g. mirtazapine) are available elsewhere [35], safer strategies to assist with sleep challenges should also consider non-medication-based options. For instance, a parallel-group trial conducted by Chakravorty et al. [36], which randomized 22 veterans recovering from alcohol dependency to receive either Cognitive Behavioral Therapy (CBT) for insomnia or monitoring, found that the CBT intervention “demonstrated substantial efficacy in reducing insomnia and improving sleepy hygiene” [36].

## Conclusion

In summary, while the widespread use of trazodone in persons with alcohol use disorder is clearly a genuine effort to address the common suffering with sleep challenges among persons with alcohol use disorder, the one large placebo-controlled trial conducted in this population, a second trial examining the trazodone analogue nefazodone, and a series of studies looking at these drugs’ metabolite m-CPP, have demonstrated that trazodone may undermine patient efforts to decrease alcohol use and with potential for increased alcohol craving. In the context of the fact that most sleep challenges in this population are secondary to alcohol use [31, 32], and given the evidence from other trials suggesting potential for increased substance use with serotonergic stimulation including by trazodone or its metabolite [14–17], the common use of trazodone among persons with alcohol use disorder implies a lack of prescriber knowledge of this literature. Through greater awareness and consideration of trazodone prescribing de-implementation strategies [37] involving the implementation of safer alternative treatment approaches [33, 35, 36], improved outcomes for patients with alcohol use disorder and insomnia can be anticipated.

## Data Availability

No datasets were generated or analysed during the current study.

## Abbreviations

M-CPP: Meta-Chlorophenylpiperazine
CBT: Cognitive Behavioural Therapy
